# Hydration-Controlled Charge Transport in Polymer Networks and Hydrogels

**DOI:** 10.3390/ma19204308

**Published:** 2026-10-13

**Authors:** Karina Mihilewicz, Katarzyna Bialik-Wąs, Konstantinos N. Raftopoulos

**Affiliations:** 1 PK KUT Doctoral School, Faculty of Chemical Engineering and Technology, PK Krakow University of Technology, Warszawska 24, 31-155 Kraków, Poland; 2 Faculty of Chemical Engineering and Technology, PK Krakow University of Technology, Warszawska 24, 31-155 Kraków, Poland; katarzyna.bialik-was@pk.edu.pl

**Keywords:** hydrogel electrolytes, water dynamics, ion transport, thermoresponsive polymers, bioelectronics, percolation theory, organohydrogels

## Abstract

Water in soft electrochemical materials is far more than a passive solvent—it directly governs ionic transport, dielectric response, and structural stability. Unlocking the full potential of hydrogel electrolytes requires mastering the delicate interplay between polymer networks, confined water dynamics, and mobile ions. Classical models like Maxwell–Garnett fall short in capturing these complex interfaces; percolation-based frameworks like General Effective Medium (GEM) theory can describe transport transitions, though their parameters remain system-dependent. This review bridges molecular-level hydration phenomena, including non-freezing bound water, organohydrogel hybridization, and thermoresponsive phase behavior, with macroscopic performance metrics across bioelectronics, energy storage, and electronic skin. By integrating percolation physics, molecular design, and quantitative cross-system comparisons across material systems, we provide a roadmap to engineer resilient, high-performance soft electroactive architectures for next-generation technologies.

## 1. Introduction

Hydrogels are three-dimensional, cross-linked polymeric matrices characterized by their ability to absorb and retain vast amounts of water while maintaining their structural integrity. Due to their high water content, these materials are soft, flexible, and have a low Young’s modulus, which allows them to mimic the mechanical properties of soft biological tissues [1,2]. This mechanical compatibility, combined with the fact that hydrogels can conduct ions in a manner analogous to that of body fluids and the extracellular matrix, has made them a fundamental building block of modern bioelectronics, soft robotics, and flexible wearable sensors [1,3].

However, the ionic conductivity of a hydrogel is not a simple function of its total water content alone. It depends jointly on the physical state of that water, specifically the distribution of free, intermediate, and strongly bound fractions, as well as the segmental mobility of the surrounding polymer chains, the chemistry of both the polymer and the mobile ions, and the morphology of the hydrated network [4,5]. Disentangling how these coupled variables jointly determine charge mobility is therefore a prerequisite for the rational, predictive design of hydrogel-based devices (Figure 1).

The water trapped in these polymer networks does not merely play a passive role as a solvent or filler. It is an active participant in biochemical and physicochemical processes, and the current literature increasingly treats it as a structural and dynamic probe of the surrounding polymer network [4,6]. It is precisely the dynamics, distribution, and degree of confinement of water molecules that provide direct information about the local macromolecular environment and the architecture of the polymer network itself [5,6]. Water determines the degree of hydrogel swelling, its mechanical stability, and, most importantly, the transport mechanisms of small molecules, nutrients, drugs, and charge carriers, including ions or electrons [4,5,7,8].

Despite the immense importance and rapid development of materials engineering, there is still a lively debate in the literature regarding the molecular nature of trapped water and its direct impact on macroscopic transport phenomena [5,6,9]. While it is known that water drastically alters the plasticity and conductivity of polymers, the mechanisms by which different states of hydration facilitate or inhibit charge mobility are interpreted in various ways [5,9]. Previous reviews have examined hydration and polymer dynamics [5], ionic conduction and hydrogel applications in bioelectronics [1], and mixed ionic–electronic transport in polymers [10]. These contributions provide complementary foundations for understanding hydrated electroactive materials. The present review connects these perspectives by treating hydration as a state variable linking local water environments, polymer relaxation, aqueous-domain connectivity, and collective charge transport. Its focus is on when water-state descriptions and transport models are useful, what experimental evidence supports their mechanistic interpretation, and where conclusions remain system-dependent. Quantitative examples are used to relate these mechanisms to the competing requirements of conductivity, mechanical integrity, and long-term stability in practical devices. To this end, we first examine the thermodynamic states and relaxation dynamics of confined water within polymer networks (Section 2) and its coupling to polymer segmental dynamics (Section 3). Building on this foundation, we analyze the specific mechanisms governing purely ionic transport (Section 4) and mixed ionic–electronic conduction (Section 5). We then translate these molecular mechanisms into actionable design rules across bioelectronics, energy storage, and flexible sensors (Section 6). Finally, Section 7 synthesizes recurring theoretical controversies, addresses long-term stability bottlenecks, and outlines emerging computational paradigms, including machine-learning interatomic potentials.

## 2. Hydration as a State Variable in Polymer Networks

To understand transport in hydrogels, we must first define the physical form in which the solvent exists within them. Water absorbed by polymers interacts strongly with the matrix, and therefore rarely constitutes a physically and thermodynamically homogeneous environment [2,4,5,9].

### 2.1. Competing Models of Water States

Historically, based on early studies of swelling phenomena and calorimetry, Jhon and Andrade proposed a classical three-state model of water in hydrogels [5,11]. According to this approach, which is popular in the literature, the water within the polymer network is divided into three distinct categories with different degrees of mobility (Table 1). It is worth stressing from the outset that these categories are best understood as operational classes along a continuum of binding strength and mobility, rather than as sharply separated physical species: their apparent boundaries depend strongly on the method and timescale of observation used to identify them (Section 2.2):

- Non-freezing water (strongly bound): It consists of water molecules that form direct, strong hydrogen bonds or electrostatic interactions with the hydrophilic groups of the polymer (e.g., hydroxyl, carboxyl, or sulfonate groups). These molecules are so firmly anchored in the matrix that they behave thermodynamically and dynamically as part of the polymer chain. Due to the drastic reduction in mobility, this fraction does not crystallize even upon cooling to 200 K;
- Intermediate water (bound freezing water): This includes molecules that interact more weakly with polymer chains or bind to the first layer of non-freezing water. This fraction exhibits melting and crystallization properties; however, due to spatial and energetic constraints, its melting point is significantly lower than that of ordinary water, a phenomenon often associated with substantial supercooling;
- Free water: It is located at the center of larger macropores, interacts only minimally with the polymer network, and behaves thermodynamically in a manner analogous to water in the free state, exhibiting a melting temperature and enthalpy consistent with those of pure water (approx. 273 K) [2,4,5,9].
- Despite the immense popularity and usefulness of this discrete model, a scientific debate continues in the literature, sparked by proponents of an alternative thermodynamic framework, discussed in several studies [4,5,12], including the review by Roorda [12]. In this view, water in the hydrogel is not divided into discrete classes at all; rather, its binding strength and mobility vary continuously with local polymer density and crosslink topology. Proponents of this framework argue that the melting and freezing behavior observed in calorimetric studies, commonly interpreted as hard evidence for distinct water ‘classes’, may instead simply reflect the kinetics of non-equilibrium freezing in a structurally graded system, rather than the existence of permanently separated confinement states. Both models provide useful insights, and the difficulty in unambiguously defining rigid boundaries between states of water stems from the fact that the relaxation time and the observed mobility of molecules depend strongly on the time scale or frequency of the analytical technique used [5,9,13].

**Table 1:** A summary of water fractions in hydrogels.

Water State (Fraction)	Type of Interaction with the Matrix	Ability to Crystallize	Melting and Evaporation	Source
Free water (bulk-like/FW/FRW)	Negligible or no direct interactions with polymer chains; retains the physicochemical properties of pure bulk water; high molecular mobility; forms continuous channels within the swollen gel.	Able to crystallize (freezable).	Melts close to 0 °C (273 K); evaporates below 100 °C.	[2,4,5,7,9,13,14,15,16,17,18,19,20,21,22,23,24]
Intermediate (weakly bound/freezable/IW/IMW)	Weak to intermediate interactions with polar groups of the matrix or with strongly bound water; forms hydration layers or clusters within polymer domains; acts as a bridging medium between chains.	Able to crystallize (freezable); frequently shows strong supercooling or cold-crystallization behavior.	Melts below 0 °C (depression caused by spatial confinement and interactions); typically –20 °C to 0 °C.	[2,4,5,7,9,13,14,17,20,22,23,24]
Strongly bound (non-freezing/BW/NFW)	Strong interactions (hydrogen bonding, electrostatic forces, dipole–dipole) directly with hydration centers (e.g., –OH, –COOH, –NH_2_, –SO_3_H groups); forms the first sorption layer; thermodynamically behaves as part of the polymer chain.	Intrinsically non-freezable. Bypasses ice crystallization even at cryogenic temperatures (down to −150 °C (123 K) or lower), undergoing vitrification into an amorphous glass state upon cooling.	Shows no measurable first-order phase transition (melting) within the accessible DSC range; evaporates above 100 °C (often around 150–200 °C).	[2,4,5,7,9,13,14,16,17,19,20,21,22,23,24,25,26,27,28]

Abbreviations: FW, free water; FRW, free-rotating/freezable water; IW, intermediate water; IMW, intermediate-bound water; BW, bound water; NFW, non-freezing water.

### 2.2. Advanced Techniques for Mapping Water Dynamics

As noted above, the boundary between these operationally defined water populations is not fixed, but depends on the specific physical phenomenon probed, the observation timescale, and the frequency window of the analytical technique used [4,5,6,9]. Each analytical technique therefore “sees” these water states, and the mobile charge carriers within them, through fundamentally distinct physical mechanisms [5,6]:

Differential Scanning Calorimetry (DSC) probes water through the thermodynamics of first-order phase transitions (melting and crystallization) [2,5], but also indirectly through plasticization effects, shifts in the polymer glass transition temperature ($T_{g}$), and distinct dynamical transitions between bound and intermediate water regimes. Free water is detected via its sharp melting endotherm near 273 K with bulk-like enthalpy (approximately 333.5 J g^−1^ for hexagonal ice) [4,7], whereas intermediate water exhibits a depressed melting temperature or cold crystallization due to confinement and steric constraints [13,29]. Strongly bound (non-freezing) water does not exhibit crystallization over the experimentally accessible temperature range and undergoes vitrification, owing to intense hydrogen bonding or electrostatic coordination with polar or charged polymer groups [4,9]. Consequently, DSC provides a dual thermodynamic map: quantifying distinct hydration fractions while revealing how bound water alters the segmental mobility of the underlying network [2,15].

Broadband Dielectric Relaxation Spectroscopy (DRS), particularly in its microwave variant, evaluates water through dipolar rotational dynamics across frequency space [6,8,9]. Free (bulk-like) water displays a characteristic dipolar relaxation near 18–20 GHz (corresponding to relaxation time τ≈8.3 ps) [6,9], whereas rotationally bound water, solvating polar or charged groups, shows restricted rotational freedom with longer relaxation times (τ>8.3 ps) shifted toward lower frequencies [5,9]. Irrotationally bound water molecules are so severely constrained that they cannot reorient with the oscillating electric field at all, remaining dielectrically invisible, a population that correlates closely with the calorimetric concept of non-freezing water [8,9]. Crucially, unlike purely thermal methods, DRS also directly observes ionic transport through the low-frequency direct-current conductivity plateau ($\sigma_{dc}$), interfacial Maxwell–Wagner–Sillars (MWS) polarization, and electrode polarization [14,15,30]. The static dielectric constant extracted from microwave DRS further serves as a key input parameter in electrostatic ion-exclusion models such as the Born and Manning frameworks [8,9].

Nuclear Magnetic Resonance (NMR) spectroscopy, encompassing spin-lattice ($T_{1}$) and spin-spin ($T_{2}$) relaxation times as well as Pulsed Field Gradient (PFG-NMR) diffusometry, probes both local reorientational dynamics and translational self-diffusion [2,5,6]. Because chemical exchange between free and bound water sites typically occurs faster than the NMR measurement window (milliseconds), PFG-NMR frequently detects a single, population-averaged self-diffusion coefficient rather than rigid, isolated fractions:

(1) $$D_{obs}=p\cdot D_{bound}+\left(1-p\right){\cdot D}_{free}$$

where $D_{obs}$ is the experimentally observed population-averaged self-diffusion coefficient, $D_{bound}$ and $D_{free}$ are the self-diffusion coefficients associated with more strongly bound and more mobile water populations, respectively, and p is the fractional population of the bound component.

This reflects the continuum nature of water mobility under confinement [6,31]. Furthermore, PFG-NMR enables direct measurements of the self-diffusion coefficients of both mobile water and individual ionic species ($D_{+}$, $D_{-}$), providing the self-diffusion coefficients that, when combined with conductivity measurements, allow deviations from Nernst–Einstein behavior and ionic correlations to be assessed, a theme we return to in Section 4.4 [32,33,34].

Vibrational spectroscopies (FTIR and Raman) detect water through the stretching modes of O–H bonds in the 3000–3600 cm^−1^ region [21,29,31]. Redshifts or blueshifts of the O–H stretching band reveal the local hydrogen-bonding architecture in detail, distinguishing isolated, single-donor/acceptor water molecules from extended hydrogen-bonded water clusters, and identifying specific ion–water coordination shells [21,29]. Simultaneously, in FTIR spectra, the strengthening or formation of hydrogen bonds induces an opposing blueshift in the O–H bending mode (near 1600–1650 cm^−1^) due to in-plane stabilization [29]. Furthermore, spectral deconvolution of the Raman O–H stretching envelope—specifically resolving the sub-bands near 3220–3250 cm^−1^ (strongly hydrogen-bonded water) and 3400–3450 cm^−1^ (weakly hydrogen-bonded water)—provides a quantitative probe for tracking how ion incorporation or matrix hydration alters the water network [21,31].

### 2.3. From Nanoclusters to Highways: The Percolation Threshold and Trapping

In the design of transport systems, both ionic conductors and drug carriers, not only the amount and binding state of water but also its spatial distribution is crucial [5,8]. At low hydration levels, sorbed water initially binds around the most hydrophilic sites of the polymer matrix (e.g., ionic groups) [21,33], forming nanoclusters that are physically separated from one another [13,14,24]. The transport of charges and molecules under such conditions is extremely difficult, as it requires molecules to “hop” across dry polymer barriers [24,25,35].

However, as the water concentration increases, the system approaches a critical point known as the percolation threshold, at which the population of isolated water clusters becomes statistically likely to interconnect into a continuous, three-dimensional network of channels spanning the polymer matrix [9,24] (Figure 2). In idealized, homogeneous systems, this transition is predicted to occur sharply, over a narrow range of water content [9]; in real hydrogels, however, structural heterogeneity, spatial gradients in local swelling, variations in crosslink density and crystallinity, and finite equilibration times typically broaden this transition into a more gradual crossover rather than a single, discrete threshold [5,32]. Despite this broadening, crossing into the percolated regime is consistently associated with a marked change in the material’s dielectric response and a multi-order-of-magnitude acceleration of ionic conductivity and diffusion, as ions gain access to continuous aqueous pathways [8,9,24].

This behavior has been documented directly in several hydrated ionomer systems. Hydration in ionomers is commonly quantified by the hydration parameter λ ($\lambda =\left[H_{2}O\right]/\left[{\mathrm{SO}}_{3}^{-}\right]$), defined as the number of water molecules per fixed ionic group; Perfluorosulfonic acid (PFSA) membranes like Nafion exhibit an early hydration onset (λ≈2−3) that reaches full structural percolation at λ≈5−6—where small-angle scattering and conductivity measurements reveal a 2-to-3-order-of-magnitude surge in proton conductivity up to ~10^−1^ S cm^−1^—sulfonated aromatic polymers require higher hydration levels to establish continuous pathways. Specifically, in sulfonated poly(ether ether ketone) (SPEEK), the percolation threshold is shifted to higher hydration levels (λ≈5−6), a direct consequence of the higher tortuosity and narrower hydrophilic domains characteristic of aromatic backbones [36,37,38]. In polyelectrolyte hydrogels based on poly(acrylic acid) (PAA) and its salts, impedance spectroscopy reveals an analogous conductivity jump, often exceeding 3 orders of magnitude, coinciding with chain ionization and the onset of a continuous aqueous phase upon swelling [4,31,39]. In PEDOT:PSS—poly(3,4-ethylenedioxythiophene):poly-(styrenesulfonate) thin films used in organic electrochemical transistors, controlled-humidity studies show that ionic conductivity rises sharply by several orders of magnitude once absorbed water forms a percolating network through the hydrophilic PSS-rich domains [40,41,42]. Understanding this sensitivity to morphology, rather than merely to total water content, allows engineers to rationally design “switchable” materials, in which a small change in moisture content can open up substantial transport pathways [41,43].

### 2.4. Hydrogel Electrolyte Design Strategies and Transport Processes

To establish a comprehensive bridge between the structural design of hydrogel networks and their ion-conducting behavior, hydrogel electrolytes can be described along several complementary design dimensions: the charge carried by the polymer, solvent composition, salt loading, incorporation of fillers, and response to external stimuli. In these soft solid-state ionic conductors, charge transport is dictated by molecular interactions established between the polymer backbone, mobile ionic species, and solvent molecules [1]. The categories outlined below represent overlapping design strategies rather than mutually exclusive material families—for example, a polyelectrolyte network may also contain nanofillers, a water–glycerol solvent mixture, and thermoresponsive segments [32]. These complementary design strategies and associated transport processes are schematically illustrated in Figure 3. Each strategy alters the concentration or mobility of charge carriers through specific molecular-level interactions between the polymer matrix, mobile ions, and solvent environment. In all systems, the overall ionic conductivity ($\sigma_{NE}$) relies on the concentration ($C_{i}$), charge valence ($z_{i}$), and effective diffusion coefficient ($D_{i}$) of the mobile ions. In the ideal case, where charge carriers are uncorrelated, it is usually described by the Nernst-Einstein relation:

(2) $$\sigma_{NE}=\frac{F^{2}}{RT}\sum_{i}z_{i}^{2}C_{i}D_{i}$$

where $\sigma_{NE}$ is the Nernst–Einstein conductivity predicted for uncorrelated charge carriers; *i* indexes the mobile ionic species; $z_{i}$, $C_{i}$ and $D_{i}$ are the charge number, molar concentration, and self-diffusion coefficient of species *i*, respectively; F is the Faraday constant, R is the universal gas constant, and T is the absolute temperature. However, the microscopic factors governing $D_{i}$ depend on the particular combination of polymer chemistry, solvent composition, and network structure [32].

#### 2.4.1. Neutral Salt-Swollen Hydrogels

Neutral salt-swollen hydrogels consist of non-ionic polymer networks, such as poly(vinyl alcohol), poly(acrylamide), or poly(ethylene glycol), swollen with aqueous salt solutions [5,44]. In these systems, charge transport is predominantly dictated by the vehicular diffusion of hydrated ions migrating through fluid-filled matrix pores [33]. The uncharged polymer backbone acts primarily as a physical barrier. This dependency can be described by Yasuda’s free-volume model:

(3) $$\frac{D_{i}}{D_{i,0}}=\exp\left(-B\frac{\phi_{p}}{1-\phi_{p}}\right)$$

where $D_{i}$ is the effective diffusion coefficient of species i in the hydrogel, $D_{i,0}$ is the ion diffusion coefficient in pure water, $\phi_{p}$ is the polymer volume fraction (corresponding to water volume fraction $1-\phi_{p}$), and B is an empirical structural parameter relating ion size to available free volume [4]. Consequently, ionic mobility is governed by free-volume availability, matrix tortuosity, and mesh size, which constrain ionic diffusion according to modified Stokes-Einstein relationships [32].

#### 2.4.2. Polyelectrolyte Hydrogels

Polyelectrolyte hydrogels incorporate polymer chains bearing covalently bound ionizable groups—either anionic (e.g., poly(acrylic acid), carboxymethyl cellulose, or alginate) or cationic (e.g., chitosan or poly(diallyldimethylammonium chloride))—counterbalanced by mobile counterions [1]. Charge transport in these systems can involve electrostatic ion hopping alongside vehicular diffusion along fixed charged sites on the polymer backbone and is strongly influenced by Donnan membrane equilibrium [45]. The fixed ionic charges provide preferential single-ion conducting characteristics by suppressing co-ion migration while facilitating counterion dissociation, with local electrostatic potential energy barriers dictating the transport rate [39,46]. Yin et al. combined experiments and simulations for hydrogels containing charged and neutral repeat units. Changing the fraction of charged monomers and crosslinking density modified counterion mobility, with different responses for $H^{+}$ and ${\mathrm{Cl}}^{-}$. This illustrates why fixed-charge concentration and network structure should be considered together when comparing polyelectrolyte hydrogel conductivity [47].

#### 2.4.3. Zwitterionic Hydrogels

Zwitterionic hydrogels feature polymer networks containing equimolar cationic and anionic moieties within the same monomer unit or network structure, typically based on betaine polymers and phosphorylcholine derivatives (e.g., poly(sulfobetaine methacrylate) [PSBMA], poly(carboxybetaine methacrylate) [PCBMA], or poly(2-methacryloyloxyethyl phosphorylcholine) [PMPC]) [1]. Ion transport in these materials is regulated by the anti-polyelectrolyte effect and dense dipolar hydration [4,45]. The tightly bound hydration shells surrounding the zwitterionic motifs screen intra- and interchain electrostatic interactions. This strongly hydrated local environment can facilitate salt-ion mobility and modify the degree of coupling between ionic transport and polymer segmental relaxation [45].

#### 2.4.4. Nanocomposite Hydrogels

Nanocomposite hydrogels integrate organic or inorganic nanofillers, such as nanoclays, MXenes (two-dimensional transition-metal carbides, nitrides, and carbonitrides), graphene oxide, metallic/metal oxide nanoparticles, or polymeric nanostructures (e.g., cellulose nanocrystals/nanofibrils, chitin nanoparticles, or conducting polymer nanofibers) into the polymeric framework [48]. Charge transport is governed by interfacial conduction pathways, altered network tortuosity, and spatial percolation dynamics [48,49]. High-surface-area nanofillers often generate space-charge layers that accelerate local ion hopping and induce continuous, highly conductive percolation networks when the filler concentration exceeds the percolation threshold [14,39]. Above a critical filler volume fraction ($\phi_{c}$), the electrical conductivity scales according to dynamic percolation theory:

(4) $$\sigma \propto {\left(\phi -\phi_{c}\right)}^{t}$$

where σ is the effective conductivity, ϕ is the nanofiller volume fraction, and t is the conductivity critical exponent, whose value depends on dimensionality and network topology [37,50].

#### 2.4.5. Organohydrogels

Organohydrogels utilize binary or multicomponent solvent mixtures, replacing a fraction of water with organic co-solvents (e.g., glycerol, ethylene glycol) or ionic liquids, within polymeric networks typically constructed from poly(vinyl alcohol) (PVA), polyacrylamide (PAAm), poly(acrylic acid) (PAA), or natural biopolymers (e.g., gelatin or alginate) [51,52]. The transport mechanism is dictated by the bulk viscosity of the solvent mixture, its dielectric constant, and the dynamic restructuring of the ion-solvation shells [51,52]. While the co-solvent effectively depresses the freezing point and mitigates solvent evaporation, the accompanying increase in fluid viscosity and modified ion-solvent coordination generally raise the activation energy for ionic conduction relative to purely aqueous hydrogels [19,52].

#### 2.4.6. Water-in-Polymer-Salt Electrolytes (WiPSE)

Water-in-polymer-salt hydrogels extend the “water-in-salt” electrolyte paradigm by incorporating highly concentrated salt environments into polymer networks. These systems typically incorporate salts such as lithium bis(trifluoromethanesulfonyl)imide (LiTFSI) or zinc trifluoromethanesulfonate (Zn(OTf)_2_) within resilient polymer matrices such as poly(vinyl alcohol) (PVA) or polyacrylamide (PAAm) in concentrations potentially reaching 10–20 mol kg^−1^ [31,53,54]. In these highly concentrated environments, the charge transport mechanism transitions from classic vehicular diffusion to structural hopping (Grotthuss-like or exchange-driven transport) through crowded coordination environments [31,54]. Due to the severe deficit of free solvent molecules, ionic mobility relies on the rapid, dynamic reorganization of contact ion pairs, ionic aggregates, and transient cation-polymer coordination complexes [55,56]. Charge transport becomes strongly coupled to the dynamic structural relaxation of the polymer-salt coordination complexes, often following Vogel-Fulcher-Tammann (VTF) temperature dependence:

(5) $$\sigma \left(T\right)=\sigma_{0}\exp\left(-\frac{E_{a}}{R\left(T-T_{0}\right)}\right)$$

where σ(T) is the ionic conductivity at temperature T, $\sigma_{0}$ is the pre-exponential factor, $E_{a}$ is the pseudo-activation energy, R is the universal gas constant, and $T_{0}$ is the empirical reference temperature (Vogel Temperature) lying below the glass transition temperature ($T_{g}$) of the polymer-salt matrix [33,35,37].

#### 2.4.7. Thermoresponsive Hydrogel Electrolytes

Thermoresponsive hydrogel electrolytes incorporate stimuli-responsive polymers (e.g., poly(*N*-isopropylacrylamide), poly(N-vinylcaprolactam), poly(N,N-diethylacrylamide), poly(2-(dimethylamino)ethyl methacrylate), poly(methyl vinyl ether)) that exhibit lower or upper critical solution temperature (LCST/UCST) phase behavior (Table 2) [1,13,57]. In LCST-type systems, heating induces an entropy-driven phase separation where polymer chains collapse as hydration shells are disrupted. Conversely, UCST-type systems undergo enthalpy-driven solubilization, expanding upon heating as polymer–polymer interactions are broken [13].

Charge transport is dynamically regulated by thermally induced conformational transitions of the polymer chains (e.g., coil-to-globule transition) [13,67]. Traversing the phase transition temperature induces rapid alterations in matrix hydration, free volume, and mesh size, leading to a reversible gating or switching of the ionic conductivity [13,57].

As demonstrated across these complementary design strategies, while the intrinsic chemical design determines the dominant transport pathways, the unifying governing factor across all systems is the dynamic interplay between the polymer backbone, mobile ions, and solvent molecules. To fully elucidate the microscopic origin of these transport mechanisms, it is necessary to examine how local hydration levels directly dictate the mobility, glass transition, and structural relaxations of the polymer framework, a topic discussed in detail in the following section.

## 3. The Effect of Hydration on the Dynamics of the Polymer Matrix

The presence of water molecules within the polymer network drastically alters its dynamic landscape. The macroscopic and microscopic properties of hydrogels result from a very close, often nonlinear coupling between the dynamics of the solvent and the relaxation of the polymer backbone itself [17,29]. Critically, these structural and dynamic modifications directly govern ionic transport (σ), as charge carrier mobility is inherently coupled to both solvent dynamics and matrix relaxations [8,9,33,35,37]. Consequently, phenomena such as plasticization, $T_{g}$ depression, α-relaxation, secondary relaxations, crystallinity, phase separation, and confined water dynamics are not merely polymer-physics concepts, but the key physical factors controlling ion transport [5,13,17,35].

### 3.1. Plasticization and the “Slaving” Mechanism

The most visible and widely studied effect of introducing water into a polymer matrix is the phenomenon of plasticization. Water penetrates between the polymer chains, loosens the structure, and increases the free volume of the system, which manifests macroscopically as a dramatic decrease in the glass transition temperature ($T_{g}$). Calorimetric measurements clearly show that the initial addition of water causes a rapid, steep drop in the glass transition temperature due to the promotion of chain flexibility. This relationship is often described using classical models such as the Fox equation or the Gordon-Taylor equation [4,7,13,29]. However, these traditional models assume ideal mixing with negligible specific interactions between components. At low hydration levels, where water is predominantly bound to specific polar moieties along the polymer backbone, water directly “feels” the local chemical architecture of the polymer network [9,68]. To account for these strong specific interactions, generalized models such as the Kwei equation introduce an empirical interaction parameter (q):

(6) $$T_{g}=\frac{w_{1}T_{g1}+kw_{2}T_{g2}}{w_{1}+kw_{2}}+qw_{1}w_{2}$$

where $T_{g}$ is the glass transition temperature of the mixture, $w_{1}$ and $w_{2}$ represent the mass fractions of the polymer and water, respectively, $T_{g1}$ and $T_{g2}$ are the glass-transition temperatures assigned to the corresponding neat components in the model; k is the Gordon-Taylor fitting parameter, and q quantifies the strength of specific polymer-water hydrogen-bonding interactions [68,69]. Such specific hydration dynamics at low water contents have been prominently highlighted in studies on polar networks, including non-isocyanate polyurethanes (NIPUs) by Łukaszewska et al. [68,70].

However, recent studies emphasize that the reduction of $T_{g}$ through plasticization dominates primarily at relatively low or moderate degrees of hydration. At higher water concentrations, clusters of mobile water begin to form, and their dynamics are coupled to that of the polymer, giving rise to a phenomenon known as “slaving.” In this regime, rather than a unidirectional subordination, “slaving” reflects a transition toward collective motion where the dynamics of the polymer backbone and absorbed water molecules become intrinsically mixed [6,68]. Under these conditions, glass transition dynamics are no longer distinct for individual species; instead, mobility manifests as a collective relaxation process where the movement of any given molecule strongly depends on its immediate neighbors, directly defining the dynamic pathways available for ion migration [9,13]. There is a very interesting debate in the literature regarding the direction of this coupling; studies at very low temperatures show that water trapped in polysaccharide matrices remains mobile even at temperatures as low as −70 °C [17]. This means that the enslavement process may operate symmetrically—the dynamics of the polymer nanopores also slow down and “enslave” the absorbed water [5,17].

### 3.2. Molecular Relaxations and Interphase Phenomena

An analysis of relaxation phenomena using broadband dielectric spectroscopy (BDS) facilitates the assessment of this complex, reciprocal interaction. Hydrated polymers exhibit a whole hierarchy of relaxation processes. The primary process, known as α relaxation or the dynamic glass transition, is associated with cooperative movements of large segments of the main chain and is a direct, dynamic reflection of the macroscopic $T_{g}$ [14,17,35]. Below the glass transition temperature, the system freezes, which, however, does not mean that motion ceases entirely on a local scale. Secondary relaxations (β, γ) are then observed, which are responsible for the orientation of local dipoles, such as the movements of side groups or individual monomers. In turn, the presence of water initiates a powerful additional relaxation (often denoted as $\beta_{wet}$ or the trapped fast water relaxation ν), which is strongly dependent on the degree of matrix hydration. From an engineering standpoint, the trapping of water and the accumulation of ions at phase boundaries (e.g., between hard polymer domains and water clusters) generate the so-called Maxwell-Wagner-Sillars (MWS) polarization, which manifests itself extensively in dielectric spectra at high temperatures and low frequencies [5,13,17,35].

### 3.3. Dynamic Crossover and Vitrification of Confined Water

One of the most fascinating and still widely debated physicochemical phenomena observed in hydrogels is a specific change in the dynamics of the confined water itself (relaxation), noted around a temperature of −90 °C (183 K) [13,15,17]. Above this temperature, the water relaxes with a significantly higher activation energy than below this point, representing a dynamic crossover whose molecular origin remains a subject of intense scientific controversy [13].

Changes in the temperature dependence of water relaxation have been discussed in terms of fragile-to-strong behaviour, confinement effects and coupling to the surrounding matrix. Their interpretation depends on the relaxation process and experimental time window being examined [28,71,72]. A crossover in a relaxation-time plot therefore requires additional evidence before it can be assigned to a glass transition [28,71].

In glass-forming liquids, fragile behaviour denotes a pronounced non-Arrhenius temperature dependence of the structural relaxation time, commonly represented by the Vogel–Fulcher–Tammann equation (VFT). Strong behaviour denotes an approximately Arrhenius temperature dependence. These terms describe relaxation kinetics and do not themselves distinguish a liquid from a solid [28,35].

(7) $$\tau \left(T\right)=\tau_{0}\exp\left(\frac{B}{T-T_{0}}\right)$$

where τ(T) stands for the relaxation time at temperature *T,* $\tau_{0}$ represents the pre-exponential relaxation time, B is an activation parameter with units of temperature, and $T_{0}$ is the Vogel temperature. In this regime, the effective activation energy increases upon cooling due to cooperative structural rearrangements in the liquid’s hydrogen-bonded network [27,28]. Conversely, strong behaviour is described by the Arrhenius relation:

(8) $$\tau \left(T\right)=\tau_{0}\exp\left(\frac{E_{a}}{RT}\right)$$

where $E_{a}$ is a constant activation energy, reflecting a rigid, directionally bonded network with highly localized relaxation dynamics [14,28], and *R* is the universal gas constant. The fragile-to-strong transition (FST) around −90 °C (183 K) thus marks a fundamental kinetic crossover where confined water transitions from a high-temperature cooperative liquid regime to a low-temperature, barrier-limited solid-like regime (Figure 4) [13,27,28,71].

In contrast, Ngai, Capaccioli, and co-workers [72] interpreted this crossover as reflecting the vitrification of confined water. However, the distinction between a change in relaxation mechanism and true vitrification requires careful evaluation against calorimetric evidence and the dynamics of the host material [27,71,72].

Recently, Melillo et al. [27] have further elucidated the complexity of confined water vitrification using calorimetry and dielectric spectroscopy, demonstrating that nanoconfined water exhibits a structural glass transition ($T_{g}$) in the 170 to 200 K range, whereas the lower-temperature thermal event represents an annealing prepeak or “shadow glass transition”. This distinction is highly significant for hydrogel transport; as highlighted by Kripotou et al. [13], it is precisely the unfreezing of these local modes of water mobility around −90 °C that induces and triggers the micro-Brownian segment fluctuations (structural relaxations) of the surrounding polymer network, validating the concept of dynamic water-polymer coupling [13,73].

## 4. Water-Controlled Ion Transport Mechanisms

Together, the structural and dynamic features established in the preceding sections—including plasticization, polymer–water dynamic coupling, and percolation pathways—define the physical host medium through which charge transport occurs [9,33,37,68]. Having defined this hydrated matrix environment, we now address the specific mechanisms by which ions migrate within it [1,24]. While the presence of water in the polymer matrix is a necessary condition for ionic transport, the mechanism by which ions actually migrate through the hydrated network remains the subject of intense methodological and theoretical debate [32,33,37,74,75,76].

### 4.1. Local Mobility, Percolation, and Multicomponent Transport Models

Historically, the dominant approach to the transport of small molecules and ions in polymer matrices has been the free-volume theory, which originated in the work of Turnbull and Cohen [74] and was subsequently extended to polymer–solvent diffusion by Vrentas and Duda [75]. In this framework, the diffusion coefficient is a function of the probability of a “hole” appearing in the local matrix, one with a volume sufficient to accommodate the diffusing molecule, generated by thermal fluctuations of the polymer chain segments. This model is, by definition, purely kinetic and largely insensitive to specific chemical interactions between the diffusing species and the matrix [24,32,33,74,75].

This approach works satisfactorily under conditions of high hydration, that is, well above the percolation threshold (Section 2.3), where water forms continuous transport channels in the matrix that resemble a bulk liquid [9,24,33,37]. However, it encounters fundamental limitations in describing systems in states of low and moderate hydration (below the percolation threshold): free-volume models predict a smooth, continuous decrease in the diffusion coefficient as the water fraction decreases, whereas experimental transport measurements and dielectric modeling reveal a drastic, discontinuous drop in efficiency, highlighting the complete breakdown of continuous-medium assumptions (Figure 5) [9,33,37].

Specifically, experimental studies report a sharp, multi-order-of-magnitude collapse in transport rates as the system dries below a critical hydration level (Figure 5a) [33,37]. At the same time, heterogeneous phase modeling of relative permittivity confirms that non-percolating classical approximations, such as the Maxwell–Garnett (MG) model, which assumes isolated, non-interacting spherical inclusions embedded in a continuous matrix, fail to capture the dielectric behavior at low and moderate water volume fractions ($\phi_{w}$) (Figure 5b) [9]. This is because these classical continuous theories neglect the critical transition point where isolated water domains interconnect. Percolation-based frameworks, such as the General Effective Medium (GEM) theory, which unifies effective medium approximations with percolation scaling laws using a critical volumetric threshold ($\phi_{c}$), can accurately locate this threshold and model the subsequent discontinuous jump in permittivity and transport properties [9]. The value of $\phi_{c}$ is not universal but depends on polymer chemistry, ionic form, charge density, morphology, crosslink density, and the definition of water content. This severe discrepancy arises because, in the sub-percolation regime, purely volume-based kinetic models fail to account for the physical isolation of hydrophilic domains, intense ion-pair formation, and the highly non-ideal electrostatic barriers that dominate the under-hydrated polymer matrix [32,33].

For this reason, an increasing number of authors advocate shifting away from purely kinetic or volume-based descriptions in favor of Maxwell–Stefan thermodynamic frameworks [32,76,77]. Unlike classical Fickian approaches that assume independent ion migration in a continuous solvent, the Maxwell–Stefan formulation explicitly accounts for inter-species friction coefficients (ion–water, ion–polymer, and water–polymer interactions) in multicomponent, confined environments. Furthermore, by driving ionic flux via gradients in chemical potential rather than simple concentration, this model incorporates a thermodynamic factor (Γ) that directly captures the strong non-ideality, severe electrostatic hindrance, and ion-pair association characteristic of sub-percolated polymer networks [32,38,76].

At the same time, a line of research is developing that links ion transport directly to the structural (segmental, α) relaxation of the polymer itself, within the framework of the so-called coupling model put forward by Ngai [78]. The degree of “decoupling” (decoupling index) between ion mobility and matrix viscosity or segmental relaxation depends strongly on water content and is sometimes used as a quantitative measure of the deviation from the simple Stokes-Einstein/Walden regime [5,15,31].

### 4.2. Vehicular Transport, Hopping, and the Grotthuss Mechanism

At the molecular level, ion migration through a hydrated polymer matrix is classically conceptualized via two idealized limiting cases. In the vehicular mechanism, a solvated ion moves translationally through the medium while individual solvent molecules can enter or leave its solvation shell, and the rate of this process is approximately correlated with the local viscosity of the microenvironment, in accordance with the Stokes–Einstein relationship (Figure 6a) [10,31]. In the hopping (or structural diffusion) mechanism, the charge moves discontinuously through sequential hops between discrete coordination sites (e.g., ionic groups in the matrix or water molecules), without requiring the translation of the entire solvation shell (Figure 6b). This is the dominant mechanism precisely in the low-hydration regime described in Section 2.3, where the ion must “hop” across dry polymer barriers between isolated water nanoclusters [30,38,39]. In real polymer electrolytes, transport rarely operates in a single, pure regime; instead, it exists as a dynamic continuum between these two asymptotic limits, where the relative contribution of each mechanism scales non-linearly with the hydration level and the local dielectric screening of the network [33,37].

A specific, specialized case of hopping transport is the Grotthuss mechanism, which describes the anomalously rapid transport of a proton ($H^{+}$) through the sequential formation and breaking of hydrogen bonds along a network of water molecules [15,30,79]. Rather than requiring the physical translation of any single oxygen atom over a long distance, the excess charge defect is delocalized over a series of transient, dynamically fluctuating protonated water complexes (Figure 6c) [80,81]. At the molecular level, this structural diffusion is governed by a continuous transition between three representative protonated water configurations, which should be regarded as limiting motifs rather than as stable, distinct molecular species:

1. The Hydronium Ion ($H_{3}O^{+}$): The core protonated state exhibiting a trigonal pyramidal geometry (Figure 6c), with the excess positive charge primarily associated with the hydronium unit. Due to its strong electrostatic field, the hydronium core acts as a potent hydrogen-bond donor to surrounding water molecules [34,81];
2. The Zundel Cation ($H_{5}O_{2}^{+}$): An intermediate, highly symmetric complex where a single excess proton is shared and “shuttled” symmetrically between two neighboring water molecules ($H_{2}O\cdots H^{+}\cdots {\mathrm{OH}}_{2}$) via a short, strong hydrogen bond (Figure 6c), representing the primary transition state during proton transfer [82,83];
3. The Eigen Cation ($H_{9}O_{4}^{+}$): A larger, thermodynamic complex composed of a central hydronium core ($H_{3}O^{+}$) strongly hydrogen-bonded to three surrounding water molecules in its first solvation shell (Figure 6c) [37,81].

Reactive molecular dynamics simulations establish that the excess proton in fully hydrated environments primarily resides as a distorted Eigen-type complex [81]. The Grotthuss shuttling event proceeds via a femtosecond-scale fluctuation of the hydrogen-bond network, often termed the “special pair dance”, where the Eigen complex structurally deforms into a Zundel configuration, enabling the proton to cross the low-energy barrier to an adjacent water molecule and form a new Eigen complex [81,84]. The effectiveness of the Grotthuss mechanism depends critically on the existence of sufficiently connected and dynamically reorganizing hydrogen-bond pathways; local water wires may support structural proton transport even before a globally percolating aqueous network is established, as discussed in Section 2.3 [10,15].

**Figure 6:**
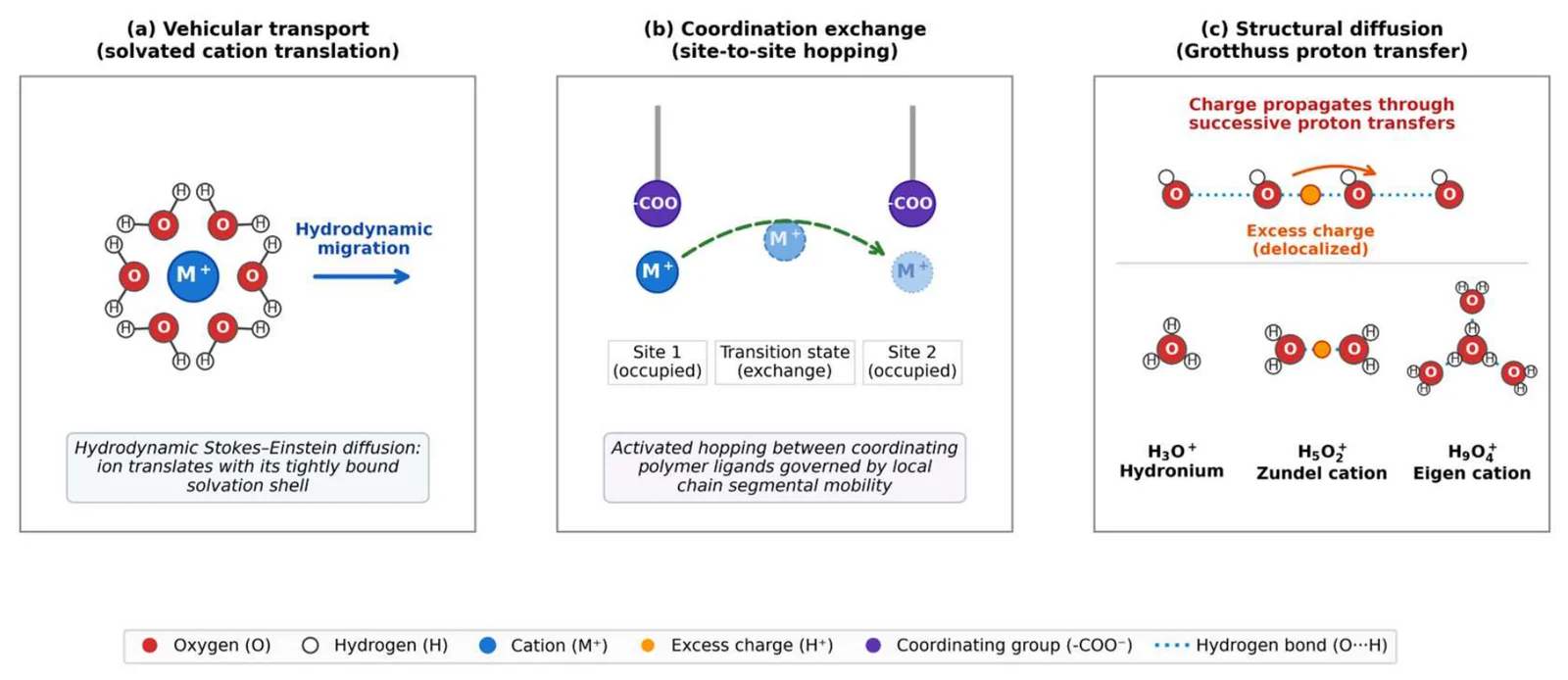
Schematic comparison of transport processes in hydrated polymer networks. (**a**) Vehicular translation of a solvated ion, accompanied by dynamic exchange within its solvation environment. (**b**) Cation transfer between coordination sites involving water and polymer functional groups. (**c**) Structural proton diffusion through proton-transfer events and reorganisation of a hydrogen-bonded water network. The lower part shows hydronium, Zundel-like, and Eigen-like motifs. These motifs represent fluctuating local configurations rather than a unique sequence of stable intermediates. Structural geometries and transition pathways are conceptually inspired by DFT energy minimization studies and reactive molecular dynamics simulations reported by Paddison and Elliot [82], Kaiser et al. [81], and Agmon [84].

A significant controversy concerns the relative contribution of these mechanisms as a function of the hydration level. Some authors, working primarily with Nafion-type membranes and related perfluorinated ionomers, point out that even at moderate hydration, local “water wires” form along the walls of the ionic channels, supporting a substantial contribution of Grotthuss transport well below full saturation [38,82,85]. Other authors, drawing on molecular dynamics simulations and measurements of the H/D isotope effect, argue that above the percolation threshold, the dominant role is assumed by vehicular transport coupled with ordinary translational water diffusion, while the Grotthuss contribution decreases as hydration increases and the environment increasingly resembles bulk water [30,38]. This discrepancy remains largely unresolved, partly due to methodological difficulties in experimentally separating the two contributions without resorting to computational modeling [30,32].

### 4.3. Water-in-Polymer-Salt (WiPSE) Electrolytes

The entire description so far implicitly assumes a classical dilute electrolyte regime, in which water molecules significantly outnumber ions, and each ion is fully surrounded by its own solvation shell composed exclusively of water [53,54]. This assumption breaks down completely under conditions of extreme salt doping, starting at molalities exceeding 5 m, where salt begins to dominate the system, and extending to ultra-high concentration regimes of 14 m to 21 m (equivalent to ≈4–10 M in molarity) [31,53,54]. At these extreme loadings, where the salt outnumbers the solvent by both weight and volume [53], the average hydration number (the mole ratio of water molecules to active cations, $n_{w}$) collapses from its classical fully solvated state of approximately 4 to 6 water molecules per ion down to as low as 2.6 for a 21 m LiTFSI system [33,53]. Consequently, the available water is insufficient to form a complete primary solvation sheath, forcing the counter-anions to penetrate the cation’s coordination shell and completely eliminating the fraction of free bulk water in the system [31,53,54]. Suo et al. [53] established the water-in-salt electrolyte concept for high-voltage aqueous lithium-ion batteries. In the context of aqueous electrolytes for batteries, this describes systems with such a high salt concentration (molality) that water, and not the salt, becomes the minor component, ensuring that nearly every water molecule remains directly coordinated to a cation or anion [31].

Applying this concept to polymer matrices gave rise to a class of materials known as Water-in-Polymer-Salt (WiPSE) electrolytes, which combine a polymer host with a superconcentrated salt solution trapped within its network [31,55,56,86]. Figure 7 compares a dilute electrolyte within a neutral polymer network (Figure 7a) with a polymer host containing concentrated molecular salt (Figure 7b). In addition to systems containing an added molecular salt, WiPSEs can also be based on a polymer salt, as illustrated in Figure 7c. In this case, the polymer chains carry fixed anionic groups balanced by mobile counterions. In these systems, the concept of “trapped water” from Section 2 undergoes a radical reinterpretation: at such extreme concentrations, the classical three-state classification into free, intermediate, and bound water (Table 1) loses its operational meaning, since virtually the entire water population exhibits a strongly bound/non-freezing character [9,33,54], and ion transport occurs through a network of directly adjacent ion-water clusters, topologically distinct from the channels described in the context of the percolation threshold for dilute systems [31,54,56].

The structural consequences of this state include the massive formation of contact ion pairs and larger ionic aggregates, a changed coordination number of the cation, a drastically reduced thermodynamic activity of water, and a broader window of electrochemical stability for the system compared to its dilute counterparts, which is important from the perspective of electrochemical applications [31]. From the perspective of ion transport, extensive ion association and aggregate formation in WiPSE lead to a significant reduction in the transport number of a single ion and to nontrivial correlation effects, which serve as a direct introduction to the discussion in Section 4.4 [32].

### 4.4. Ionic Correlations and Onsager Transport

The classical quantitative interpretation of ionic conductivity relies on the Nernst–Einstein relation, which assumes that ion migration occurs via uncorrelated, statistically independent random walks. In this ideal framework, the macroscopic ionic conductivity ($\sigma_{\mathrm{NE}}$) is directly predicted from the microscopic self-diffusion coefficients ($D_{i}^{\mathrm{self}}$) of individual charge carriers (measured via PFG-NMR or QENS) according to:

(9) $$\sigma_{NE}=\frac{F^{2}}{RT}\sum_{i}z_{i}^{2}c_{i}D_{i}^{self}$$

where F is Faraday’s constant, R is the universal gas constant, T is absolute temperature, $z_{i}$ is the valence, and $c_{i}$ is the molar concentration of species i [32,34]. In real polymer electrolytes and hydrogels, however, measured experimental conductivity ($\sigma_{\exp}$, obtained via electrochemical impedance spectroscopy) consistently deviates from $\sigma_{\mathrm{NE}}$, being substantially lower in the vast majority of systems. This discrepancy arises because self-diffusion ($D_{i}^{\mathrm{self}}$) captures all random thermal displacements of an ion regardless of its coordination state, whereas electrical conductivity ($\sigma_{\exp}$) requires net charge displacement under an applied electric field [32,33].

To quantify this deviation, two primary physical metrics are used: the Haven ratio ($H_{R}$) and the ionicity (α):

(10) $$H_{R}=\frac{\sigma_{NE}}{\sigma_{exp}},\alpha =\frac{1}{H_{R}}=\frac{\sigma_{exp}}{\sigma_{NE}}$$

where $\sigma_{exp}$ is the experimentally measured conductivity, and $\sigma_{NE}$ is the conductivity predicted from self-diffusion coefficients. Physically, for uncorrelated transport, $H_{R}=1$ (α=1). When $H_{R}>1$ (α<1), strong inter-ionic correlations are present, reducing the measured conductivity relative to the Nernst–Einstein prediction. For instance, bound neutral ion pairs (e.g., ${\left[{\mathrm{Li}}^{+}{\mathrm{TFSI}}^{-}\right]}^{0}$) contribute fully to isotopic self-diffusion ($D^{\mathrm{self}}$) but carry zero net electric current, leading to a severe overestimation of conductivity by the Nernst–Einstein equation [34,87].

A rigorous thermodynamic framework to disentangle these coupled motions is provided by Onsager phenomenological transport theory. Here, the flux of each species i ($J_{i}$) is expressed as a linear combination of the thermodynamic driving forces ($\nabla \mu_{j}$) acting on all components in the mixture:

(11) $$J_{i}=-\sum_{j}L_{ij}\nabla \mu_{j}$$

where $J_{i}$ is the flux of species *i*, $\mu_{j}$ is the chemical potential of species *j*, $\nabla \mu_{j}$ is its spatial gradient, and terms $L_{ij}$ represent the Onsager phenomenological coefficients, coupling the flux of species *i* to the thermodynamic driving force associated with species *j* [32,87]. The diagonal terms ($L_{ii}$) describe the intrinsic mobility of species i, while the off-diagonal cross-coefficients ($L_{ij}$ where i≠j) quantify cross-correlations between distinct species [32,88]. Under the conventional displacement-correlation definition, cation–anion motion in the same direction contributes positively to $L_{+-}$. Because the ions carry opposite charges, this contribution reduces net charge transport; consequently, a negative contribution to conductivity must not be confused with a negative cation–anion displacement correlation. Meanwhile, $L_{+w}$ and $L_{-w}$ govern ion–water momentum coupling. This latter coupling contributes to electroosmotic drag, the physical phenomenon whereby migrating ions entrain water molecules along their path, treating water convection as an intrinsic component of the thermodynamic transport matrix rather than a secondary side effect [37].

A major scientific debate in this area concerns how the magnitude of these cross-correlations scales with hydration. Ganesan and co-workers [32], alongside Sujanani et al. [33], working primarily under low to moderate hydration conditions near the percolation threshold (Section 2.3), suggest that extensive ion pairing and cluster aggregation, combined with joint vehicular transport of ions with constrained solvation shells (Section 4.2 and Section 4.3), enhance $L_{+-}$ cross-correlations, causing $H_{R}$ to peak well above unity. Correlations can also enhance collective transport in other electrolyte classes, as demonstrated by Nürnberg et al. [87]. In contrast, Kusoglu and Weber [37], Bannon et al. [9], and Knauth et al. [38], studying highly hydrated systems well above the percolation threshold, observe that cross-correlations rapidly decay as solvent shielding increases. In this high-hydration limit, the Haven ratio asymptotically approaches unity ($H_{R}\to 1$), restoring the validity of the simple uncorrelated Nernst–Einstein description. This demonstrates that the Haven ratio is not a static material constant, but a highly non-linear function of local matrix hydration and dielectric screening [9,32,37].

A promising pathway toward resolving this dispute lies in advanced computational methods—specifically ab initio molecular dynamics (AIMD) simulations and machine-learning interatomic potentials (MLIPs). These atomistic frameworks enable the direct extraction of individual $L_{ij}$ cross-coefficients from equilibrium fluctuation-dissipation integrals, bypassing the need for indirect experimental approximations [32,81,88,89]. This computational approach will be revisited in Section 7 in the context of artificial intelligence in transport modeling.

## 5. Electronic and Mixed Ionic–Electronic Conduction

The mechanisms described in Section 4 concerned purely ionic transport. However, in organic mixed ionic–electronic conductors (OMIECs), this picture is expanded: ions are transported through hydrated domains, while electrons or holes move along a delocalized, π-conjugated polymer backbone [10,42,79]. In benchmark systems such as PEDOT:PSS and polyaniline (PANI), water absorption enables electrolyte ions to penetrate deep into the bulk material rather than remaining restricted to the interface [3,79,90]. The performance of these materials is often quantified using the figure-of-merit $\mu C^{*}$, defined as the product of electronic mobility (μ) and volumetric capacitance ($C^{*}$) [91,92,93].

A central controversy in mixed-conducting hydrogels lies in the opposing effect of hydration on electronic versus ionic transport pathways. While swelling inherently promotes ionic percolation (Section 2.3), the absorption of water increases intermolecular spacing, introduces structural disorder, and reduces the density of conjugated conduction paths, thereby suppressing electronic mobility (μ) [10,41,91]. Conversely, moderate hydration occasionally enhances long-range order within conductive domains via phase reorganization, resulting in a non-monotonic evolution of the figure of merit $\mu C^{*}$ with water content [40,41,94]. Consequently, an intermediate hydration regime may provide the best compromise between ionic access and electronic transport, although the optimum is strongly material-dependent [10,41].

An alternative approach to mixed conductivity relies on embedding rigid carbon fillers (graphene, carbon nanotubes) into the hydrogel matrix, creating two interpenetrating percolation networks [48,49,95]. In these carbon composites, hydrogel swelling similarly presents a double-edged effect: while beneficial for ionic percolation, matrix expansion increases the average junction distance between carbon nanostructures, risking the disruption of the electronic percolation pathway [48,95,96].

## 6. Impact and Applications

The ion and electron transport mechanisms detailed in Section 4 and Section 5, i.e., percolation, Grotthuss hopping, moisture-controlled self-doping, and interpenetrating networks, are not merely theoretical constructs [10,32,37]. They establish the direct molecular design rules for soft electrochemical and bioelectronic devices [42,79,97]. This section outlines how performance requirements across three major application domains are governed by hydration dynamics and transport trade-offs [10,42]. Table 3 summarizes the functional requirements, primary transport bottlenecks, and corresponding molecular hydration handles for each major application domain.

Water-retention strategies introduce further trade-offs. Polyols and concentrated ionic environments can reduce drying or freezing while also changing viscosity, solvation, and ion association. For wearable devices, retention should be assessed together with baseline drift and response reproducibility during mechanical cycling. For implanted devices, swelling, leaching and changes at the tissue interface are relevant in addition to evaporation. Meaningful long-term comparisons require the test duration, temperature, surrounding medium or relative humidity, and encapsulation conditions to be reported [51,52,97].

To facilitate quantitative comparison, Table 4 collates representative hydrated polymer electrolytes together with their composition, hydration level, ionic conductivity and measurement temperature, activation energy, and intended application.

The representative conductivities span approximately four orders of magnitude, from 10^−2^ to 10^2^ mS cm^−1^. However, direct ranking is inappropriate because polymer composition, hydration state, salt concentration, and measurement temperature differ substantially among the systems. The comparison also reveals a persistent reporting gap: activation energies and even conductivity measurement temperatures are unavailable for several application-oriented materials.

### 6.1. Bioelectronics and Ion-Guided Drug Delivery

- Functional Requirement: Bioelectronic interfaces, such as organic electrochemical transistors (OECTs) for neural/cardiac signal recording and organic electronic ion pumps (OEIPs) for targeted drug delivery, require high signal amplification (transconductance, $g_{m}$) and precise, millisecond-scale ionic dosage without generating convective fluid flow [1,42,97];
- Underlying Transport Bottleneck: In OECTs, achieving high volumetric charge storage generally requires sufficient matrix swelling to accommodate migrating electrolyte ions [79,94]. However, as established in Section 5, water uptake increases intermolecular spacing and structural disorder along the conjugated polymer backbone, creating a direct trade-off between ionic capacity ($C^{*}$) and electronic mobility (μ) [41,91,94]. In OEIPs, high ionic transport rates are degraded by parasitic background ion competition and strong inter-ionic correlations (Section 4.4), which lower the effective Faraday efficiency below theoretical limits [32,42];
- Design Strategy: Device performance is optimized by tuning the molecular figure-of-merit $\mu C^{*}$ via micro-phase segregation [10,79]. In PEDOT:PSS and glycolated polymers, controlling the ratio of hydrophilic (ion-transporting) to hydrophobic (electron-conducting) domains maintains electronic connectivity while permitting bulk ion uptake [40,90,91]. For OEIPs, reducing the matrix hydration level near the ionic percolation threshold ($\phi_{c}$, Section 2.3) suppresses solvent convection and enhances permselectivity for target therapeutic ions [32,42]. Specifically, Chen et al. [105] used molecular dynamics to examine ion transfer from a PVA hydrogel into PEDOT:PSS. Their analysis connected interfacial coordination changes to the design of a PVA/PSBMA electrolyte that reduced the reported OECT response time by 46% relative to a pristine-PVA hydrogel device. This example shows why bulk conductivity should be evaluated together with interfacial ion access and device response.

### 6.2. Soft Energy Storage and Osmotic Power Generation

- Functional Requirement: Aqueous batteries and supercapacitors require suitable electrochemical stability, low internal resistance, and mechanically reliable electrolytes. Reverse-electrodialysis membranes instead require a balance between ion selectivity and electrical resistance [9,53,54,76,86];
- Underlying Transport Bottleneck: In dilute aqueous hydrogels, the abundance of thermodynamic “free” bulk water induces parasitic water-splitting reactions at modest voltages (≈1.23 V), severely limiting energy density [31,53,54]. In RED membranes, excessive swelling collapses the Donnan exclusion potential, allowing co-ion leakage and compromising power conversion efficiency [9,32,76].
- Hydration-related design strategy: In aqueous batteries, the electrochemical window is expanded by shifting into the Water-in-Salt (WiSE) regime (Section 4.3). In concentrated aqueous electrolytes, altered solvation and the formation of protective electrode interphases can extend the practical electrochemical stability window. The accessible voltage range depends on the electrolyte, electrode materials, and measurement protocol, and should not be predicted solely from a water-to-cation ratio [31,53,54,55,56]. In RED membranes, the trade-off between permselectivity and resistance depends jointly on hydration, fixed-charge density, channel dimensions, ion partitioning, and local water mobility [4,9,32,76].

### 6.3. Flexible Sensors and Wearable Electronics

- Functional Requirement: Piezoresistive and capacitive sensors for electronic skin (e-skin) demand high strain/pressure sensitivity, tissue-like mechanical compliance (low Young’s modulus), and long-term signal reproducibility under ambient conditions [1,19,50,51,103];
- Underlying Transport Bottleneck: In composite hydrogels containing conductive carbon fillers (graphene, CNTs), mechanical deformation modulates the distance between conductive elements, altering resistance [48,49,50]. However, ambient water evaporation induces continuous dimensional shrinkage and matrix stiffening, triggering severe baseline drift and signal degradation over time [43,48,51,52,100];
- Design Strategy: Sensitivity and stability are controlled via interpenetrating percolation networks (Section 5) combined with solvent-retention engineering [49,50,51,100]. Hybridizing hydrogels with non-volatile ionic liquids or polyols (forming organohydrogels) can reduce solvent loss and extend the period of stable operation, depending on composition, ambient conditions, and encapsulation, while preserving soft segmental dynamics and stable percolation pathways during repeated mechanical cycling [51,52,100,103,104].

## 7. Future Perspectives

The preceding sections have established a coherent cause-and-effect continuum: from the molecular nature of confined water and its thermodynamic states (Section 2), through its coupling with polymer matrix dynamics (Section 3) and the mechanisms of ionic (Section 4) and mixed ionic–electronic transport (Section 5), to the specific architectures of bioelectronic and energy storage devices (Section 6). The present section closes this narrative chain by synthesizing recurring theoretical tensions and identifying critical areas where the current state of knowledge remains insufficient—both from a methodological and a materials design perspective.

### 7.1. Synthesis: Recurring Controversies

The discussion in Section 2, Section 3, Section 4, Section 5 and Section 6 reveals three recurring but conceptually distinct tensions operating at different physical levels: the representation of hydration states, the microscopic mechanism of charge migration, and the role of collective ionic correlations.

1. Discrete Three-State Models vs. Continuous Energy Distributions: DSC characterization partitions water into three discrete states (non-freezing, freezable bound, free water). Conversely, dielectric spectroscopy (BDS) and molecular dynamics demonstrate continuous distributions of relaxation times and hydrogen-bonding energies [5,9,12]. While discrete models offer practical operational utility, continuous frameworks are necessary to model transport kinetics near interfaces;
2. Structural and vehicular contributions: Both can contribute to proton transport, with a balance determined by the material and its hydration state. A change in conductivity with water content does not by itself identify the dominant microscopic mechanism [83,84,106].
3. Uncorrelated Nernst–Einstein vs. Onsager Cross-Correlations: The widespread use of the Nernst–Einstein equation assumes independent ion motion. In dense hydrogels and WiSE systems, conductivity ($\sigma_{\exp}$) severely diverges from $\sigma_{\mathrm{NE}}$, requiring the explicit consideration of Onsager cross-coefficients ($L_{+-}$, $L_{+w}$) to account for momentum exchange and electroosmotic drag [32,87,107].

### 7.2. Long-Term Stability: Biofouling and Degradation

The bioelectronic applications described in Section 6.1 implicitly assume the temporal stability of the material–tissue interface. However, exposing the hydrogel to its target environment undermines this assumption on two independent fronts: biological and physicochemical. Contact with physiological fluids inevitably leads to the adsorption of proteins, lipids, and cells onto the material surface—a phenomenon collectively referred to as biofouling [45,97]. Mechanistically, a material’s susceptibility to biofouling is inversely correlated with the thickness and density of the strongly bound, non-freezable water layer on its surface (Section 2.1). This hydration layer acts as a physical and entropic barrier that hinders non-specific protein adsorption, consistent with the mechanism well-established for zwitterionic antifouling materials [4,45]. Consequently, biofouling directly degrades the transport functions described in Section 4, Section 5 and Section 6: it blocks ion access to the active surface of OECTs (Section 6.1), increases the impedance of the electrode–tissue interface, and distorts the signal acquisition of flexible sensors (Section 6.3) [42,45,97].

Independent of biofouling, the polymer matrices themselves undergo water-dependent aging processes: hydrolysis of cross-linking bonds, leaching of non-covalently bound ionic or conductive dopants (particularly significant for PSS in PEDOT:PSS, Section 5), and irreversible reorganization of the domain morphology driven by swelling–deswelling cycles [50,98]. In sensor (Section 6.3) and energy (Section 6.2), applications, however, water evaporation under conditions of incomplete device encapsulation remains a major practical failure mechanism. This process leads to a progressive loss of ionic conductivity as the system recedes below the percolation threshold (Section 2.3), accompanied by a drift in electrical parameters [40,43]. Strategies for mitigating both modes of degradation—biological and physicochemical—partially overlap: surface modifications that enhance the hydration layer (to combat fouling) and barrier encapsulation or increasing the salt concentration in the spirit of WiPSE (Section 4.3, to combat evaporation) represent distinct engineering pathways that are rarely considered synergistically within a single material [45,54]. Systematic, long-term studies spanning multiple months, under in vivo or simulated conditions that combine both degradation modes remain uncommon in a body of literature currently dominated by the characterization of freshly prepared materials [50,97]. Only in the most recent literature has a new paradigm of ‘extreme hydrogel bioelectronics’ emerged, asserting the absolute necessity of simultaneously engineering materials resistant to dehydration/swelling (anti-drying/anti-swelling) and foreign body response (anti-FBR) [99]. A promising pathway to fulfill this requirement involves multiphasic organohydrogels, where coexisting organic and aqueous domains synergistically suppress solvent evaporation and protein adsorption [103]. Concurrently, a new generation of volume-stable, non-swelling zwitterionic hydrogels has demonstrated unprecedented long-term immunoprotective viability in vivo [108].

### 7.3. Transport Anisotropy

The vast majority of the theoretical models and experimental data discussed in Section 2, Section 3, Section 4 and Section 5 implicitly assume material isotropy—uniform transport properties across all spatial directions. This assumption is systematically violated in real-world hydrogels possessing an oriented microstructure: materials fabricated via directional freezing, subjected to repeated mechanical stretching, or featuring a naturally anisotropic fibrous architecture (e.g., cellulose- or silk-based hydrogels) [56,101]. In such systems, the diffusion coefficients of ions and water, as well as the electronic conductivity of the percolated carbon network (Section 5), can differ by an order of magnitude or more between the directions parallel and perpendicular to the microchannel orientation axis. This anisotropy is not merely a measurement complication; rather, it constitutes a potentially highly useful design parameter—enabling, for instance, directional routing of ionic signals in sensors (Section 6.3) or selective transport enhancement along a preferred axis in RED membranes (Section 6.2) [32,56,101]. However, systematic theoretical frameworks quantitatively linking fabrication parameters to transport anisotropy remain fragmented. Furthermore, most macroscopic models presented in Section 4 (including Onsager relations), in their standard scalar formulations, fail to explicitly account for the fundamentally tensorial nature of the transport coefficients required to accurately describe such ordered media [32,109].

### 7.4. The Role of AI and Machine Learning

A recurring methodological motif throughout this review is the fundamental limitation of classical experimental techniques in resolving local, dynamic transport kinetics:

- PFG-NMR provides isotopic self-diffusion coefficients ($D_{i}^{\mathrm{self}}$) averaged over macroscopic time and space, but cannot differentiate between net charge-carrying free ions and non-conducting neutral ion pairs;
- Broadband Dielectric Spectroscopy (BDS) is heavily masked at low frequencies by electrode polarization and interfacial Maxwell–Wagner–Sillars dispersions, obscuring intrinsic bulk ion relaxation.
- Differential Scanning Calorimetry (DSC) detects macroscopic thermal phase transitions such as freezing, melting, and glass transition-related changes but lacks the femtosecond temporal resolution needed to directly observe hydrogen-bond network reorganizations.

To bridge these experimental gaps, ab initio molecular dynamics (AIMD) simulations have been employed (Section 4.4). However, AIMD is computationally demanding and therefore remains restricted to small system sizes (<500 atoms) and short timescales (<100 ps), precluding its application to fully swollen polymer networks [81,89]. A major methodological breakthrough has been the development of machine-learning interatomic potentials (MLIPs). Trained on quantum mechanical (DFT) data, MLIPs achieve near-DFT accuracy at a fraction of the computational cost [83,88]. This enables large-scale atomistic simulations of polymer–water–ion systems sufficient for the direct, explicit determination of the full matrix of Onsager cross-coefficients ($L_{ij}$) and percolation dynamics (Section 2.3) without relying on phenomenological assumptions [24,88].

Machine-learning models can support the screening of polymer compositions by predicting thermal properties within the chemical and compositional ranges represented in their training data. Such predictions complement measurements but do not independently identify the water populations responsible for overlapping thermal events. Extending these approaches to hydrated networks requires datasets that resolve water content, network structure, and measurement conditions [110,111].

### 7.5. Concluding Remarks

Charge transport in hydrated polymer networks is governed jointly by water content, local polymer–water–ion interactions, network connectivity, and the coupling between molecular motions. Water-state classifications provide useful operational descriptions, but they do not uniquely determine ion mobility. Likewise, neither a single percolation threshold nor a universal transition between hopping and vehicular transport applies across all hydrated polymers. Relating transport mechanisms to device performance requires measurements of water uptake, conductivity, and molecular dynamics under comparable conditions. For practical hydrogel electrolytes, improvements in conductivity must be evaluated alongside mechanical integrity, water retention, and stability during use. Combining controlled-hydration experiments with complementary spectroscopy and validated molecular simulations can distinguish local mobility from collective charge transport and identify the hydration range appropriate for each application.

## Figures and Tables

**Figure 1:**
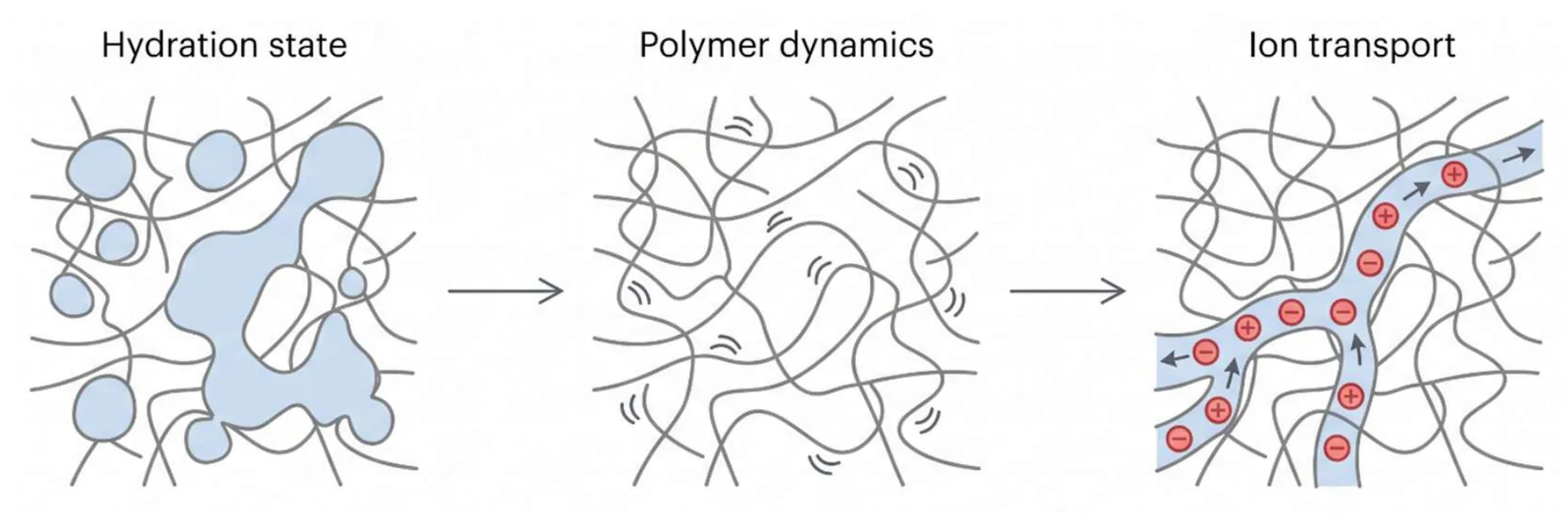
Logical structure of this review. Hydration controls charge transport by modifying polymer segmental dynamics and by changing the connectivity and mobility of water-rich domains. At low-to-moderate hydration, transport can remain strongly coupled to matrix relaxation, whereas at higher hydration, increasingly connected aqueous pathways can support more solvent-like ion motion. In the last panel, arrows indicate the direction of ion motion along the aqueous pathway.

**Figure 2:**
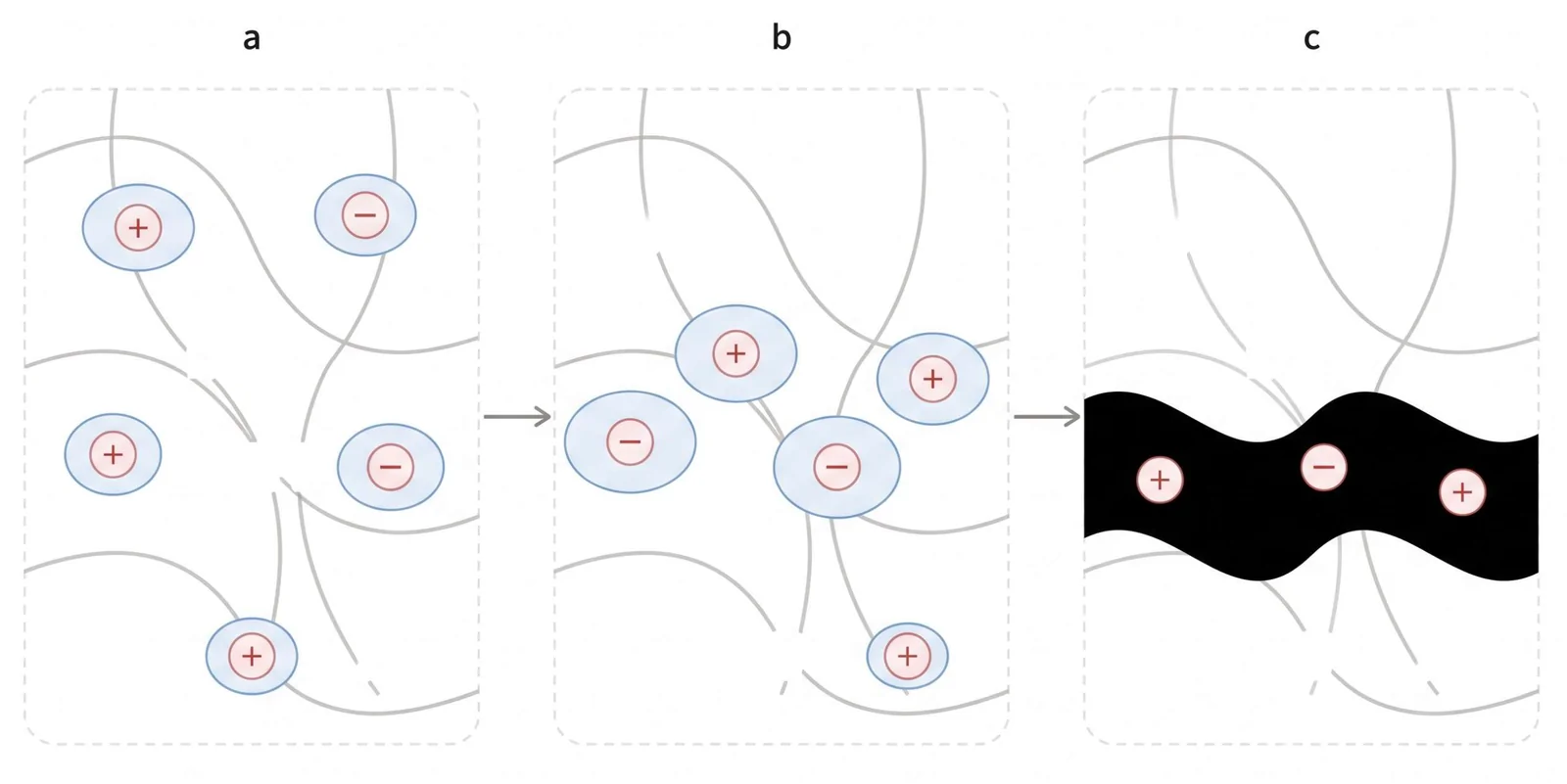
Percolation of trapped water controls ion transport. (**a**) At low hydration, water forms isolated clusters around hydrophilic sites, forcing ions to hop between them across dry polymer barriers. (**b**) Near the percolation threshold, clusters begin to touch, creating the first continuous pathways. (**c**) Above the threshold, a continuous aqueous channel spans the matrix, allowing ions to migrate largely independently of polymer segmental motion.

**Figure 3:**
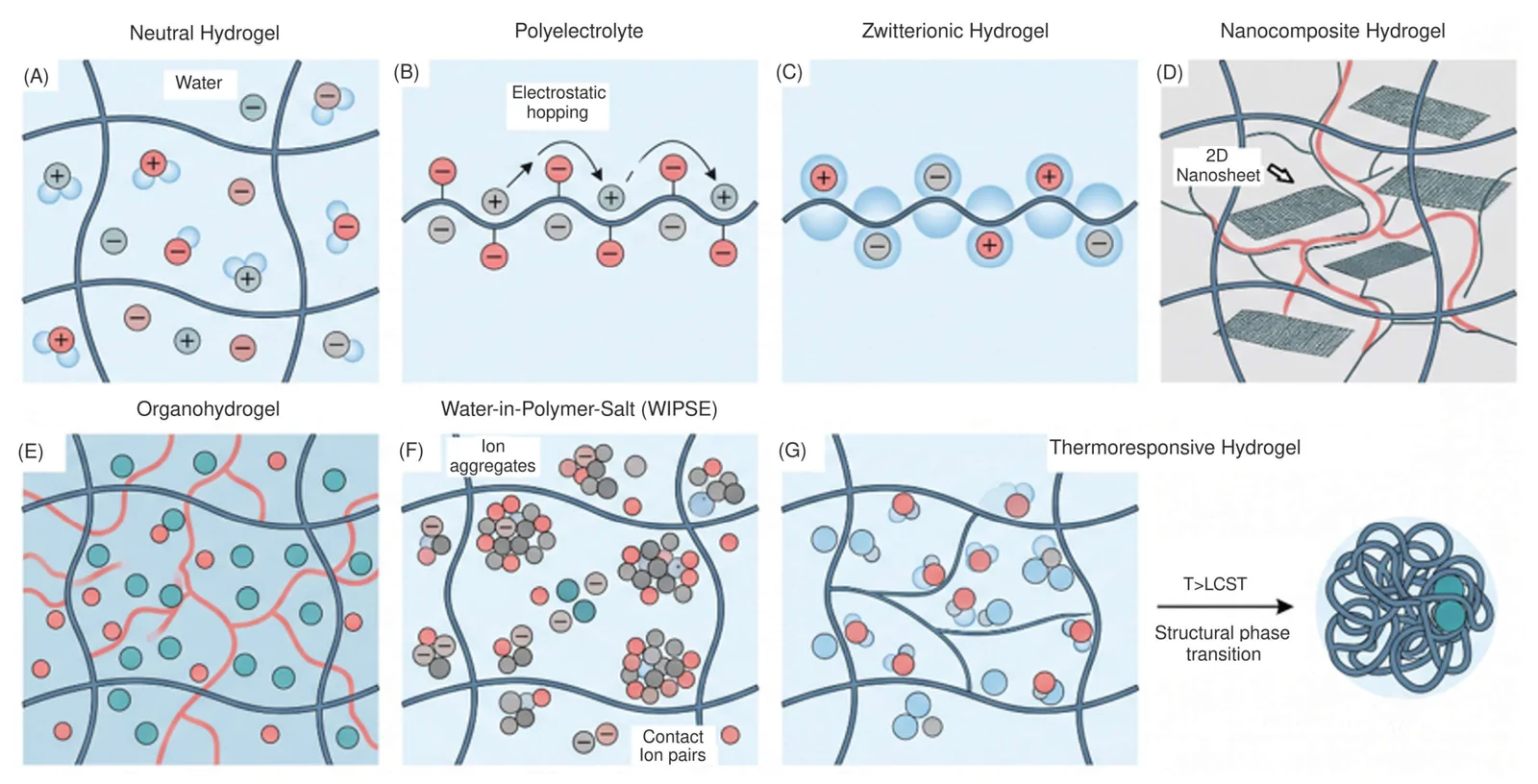
Schematic overview of complementary design strategies for hydrogel electrolytes and representative charge-transport processes: (**A**) vehicular ion diffusion through fluid-filled matrix pores in neutral salt-swollen hydrogels; (**B**) electrostatic hopping of counterions along fixed ionizable groups in polyelectrolyte networks; (**C**) dipolar hydration-mediated, low-friction transport in zwitterionic hydrogels; (**D**) interfacial conduction and percolation pathways facilitated by 2D nanosheets in nanocomposite hydrogels; (**E**) viscosity-constrained ion migration in organohydrogels with binary solvent mixtures (e.g., water/glycerol); (**F**) structural hopping through contact ion pairs and dense ion aggregates in high-salt water-in-polymer-salt systems (WiPSE); and (**G**) thermally gated ionic transport in thermoresponsive hydrogels undergoing a structural phase transition (coil-to-globule collapse) above the lower critical solution temperature (LCST).

**Figure 4:**
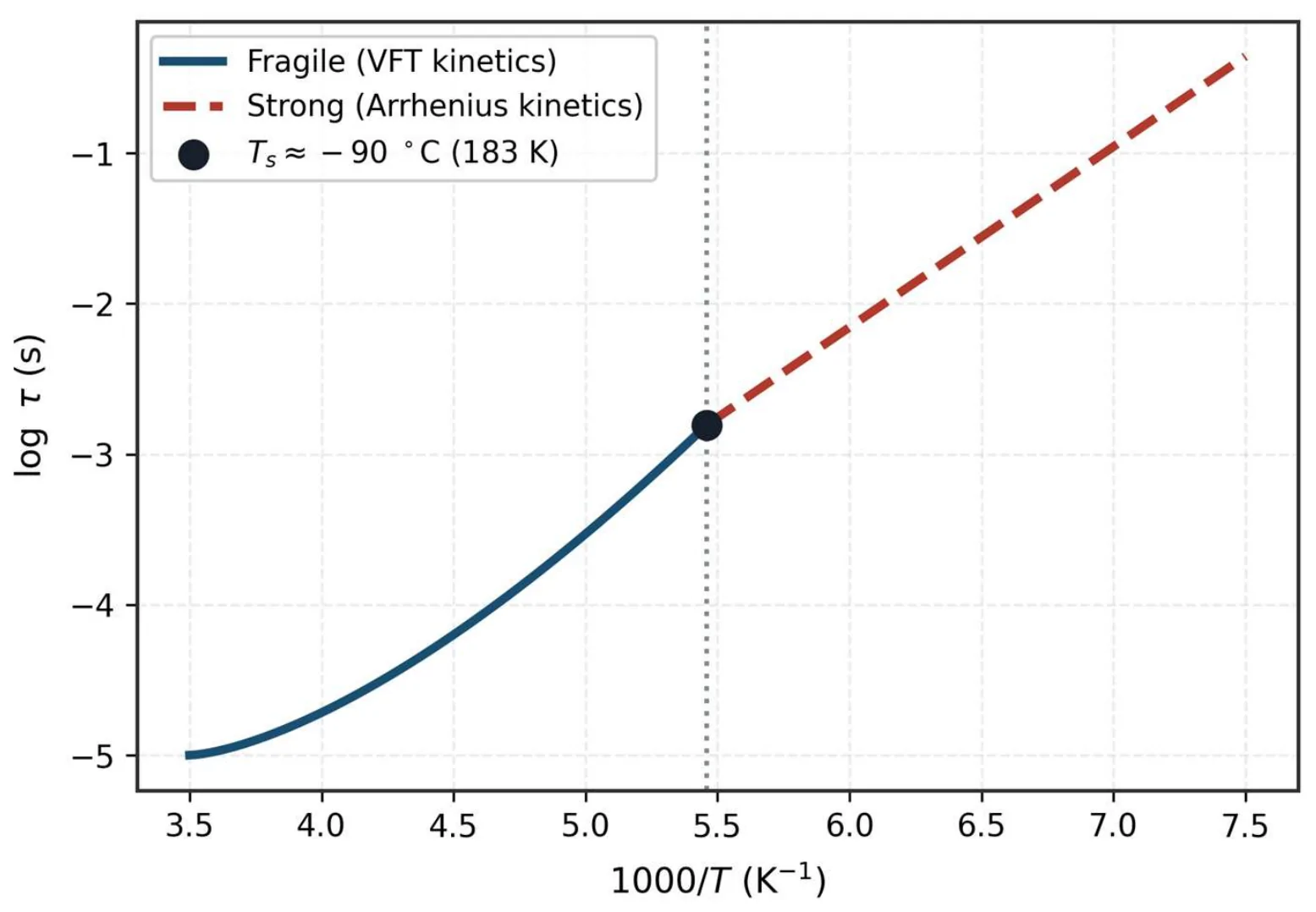
Schematic reciprocal-temperature relaxation plot (log(*τ*) vs. 1000/T) illustrating the fragile-to-strong dynamic crossover (ν-relaxation) of nanoconfined water at T ≈ −90 °C (183 K). The high-temperature region follows non-Arrhenius Vogel-Fulcher-Tammann (VFT) kinetics characteristic of a fragile liquid, whereas the low-temperature region transitions to linear Arrhenius behavior typical of a strong, directionally bonded network.

**Figure 5:**
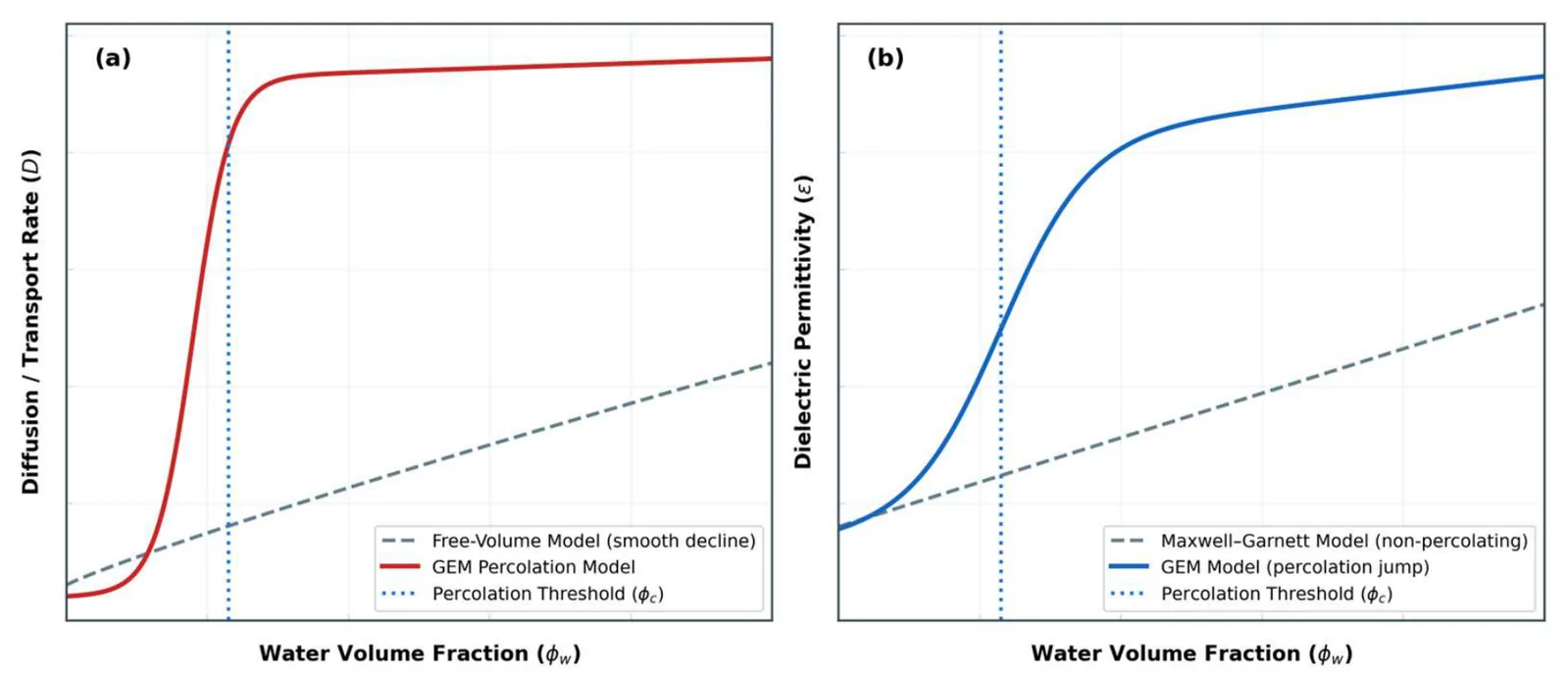
Conceptual comparison illustrating the breakdown of classical continuous and free-volume model predictions in hydrated polymer electrolytes below the percolation threshold. (**a**) Qualitative representation of the water diffusion/transport rate (D) as a function of water volume fraction ($\phi_{w}$), showing the sharp, multi-order-of-magnitude transport collapse below the critical percolation threshold ($\phi_{c}$) compared to the smooth, continuous decline predicted by classical free-volume models. (**b**) Dielectric response (static permittivity, ε) comparing non-percolating continuum models (Maxwell–Garnett approximation, dashed line) with the General Effective Medium (GEM) framework (solid line), highlighting the sharp percolation jump occurring upon the formation of interconnected hydrated pathways. (Conceptually adapted from Kusoglu and Weber [37] and Bannon et al. [9]).

**Figure 7:**
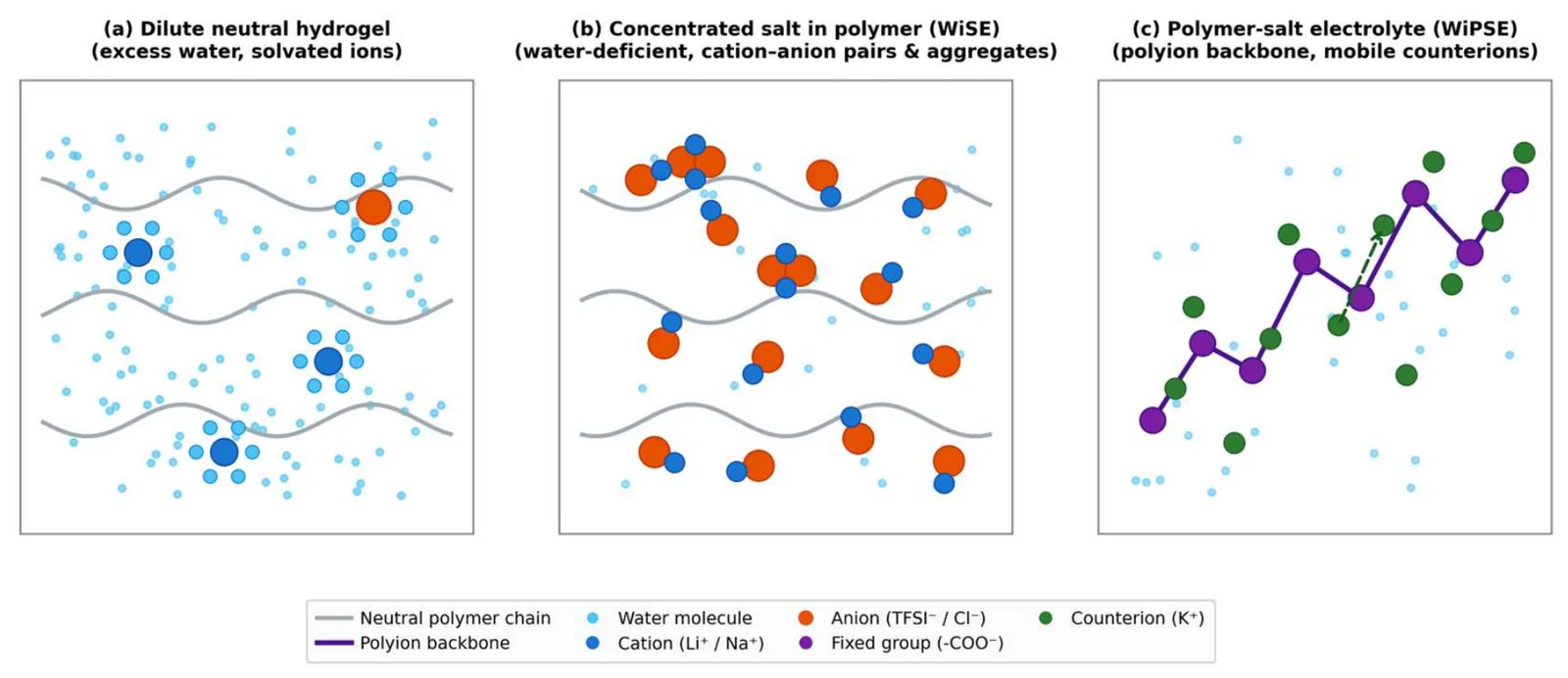
Schematic solvation environments in hydrated polymer electrolytes. (**a**) A dilute electrolyte within a neutral polymer network. (**b**) A polymer host containing a concentrated molecular salt solution, with mixed water–anion coordination environments. (**c**) A concentrated polymer-salt electrolyte containing charged chains and mobile counterions, which can hop between sites as shown by an arrow. The relative roles of water, polymer groups and ionic associations depend on formulation. The drawings indicate possible local environments and do not imply that all water is immobilised or that a single transport mechanism applies [31,53,56].

**Table 2:** Selected thermoresponsive polymers and their corresponding transition temperature values.

Thermoresponsive Polymer	Abbreviation	Typical Transition Temperature and Behavior (LCST/UCST/Sol-Gel)	Source
Poly(N-isopropylacrylamide)	PNIPAM	≈32 °C	[58]
Poly(N-vinylcaprolactam)	PVCL/PNVCL	≈30–35 °C	[59]
Poly(N,N-diethylacrylamide)	PDEAAm	≈25–32 °C	[60]
Poly(2-(dimethylamino)ethyl methacrylate)	PDMAEMA	≈50 °C	[60]
Poly(methyl vinyl ether)	PMVE	≈28–35 °C	[58,61]
Poly(2-isopropyl-2-oxazoline)	PiPrOx	≈36–39 °C	[62]
Poloxamer 407 (Pluronic F127)	P407/F127	≈20–30 °C	[63,64]
Methyl cellulose	MC	≈40–50 °C	[64,65]
Gelatin	-	≈30–35 °C	[64]
Agarose	-	≈30–40 °C	[66]

**Table 3:** Mechanistic mapping of hydration control, transport bottlenecks, and molecular design handles across key soft electrochemical and bioelectronic applications.

Parameter/Feature	Bioelectronics & Drug Delivery	Energy Storage & Harvesting	Sensors & Wearable Electronics
Key device/technology & representative polymers	• OECTs (e.g., PEDOT:PSS, glycolated polythiophenes like p(g2T-TT)) • OEIPs (e.g., qPVBC, PSSS, PVBPPh3)	• WiSE Batteries (e.g., PVA, PAAm, PEO) • RED Membranes (e.g., Nafion, SPEEK)	• e-Skin Sensors (e.g., PAAm–alginate IPNs, NaCMC, PVA/MXene hybrids) • Organohydrogels (e.g., PVA–glycerol, PAAm/IL networks)
Primary transport bottleneck	• Swelling trade-off (volumetric capacitance vs. mechanical stability) • Background ion competition & negative cross-correlations	• Narrow ESW (≈1.23 V) from free bulk water • Co-ion leakage via collapsed Donnan exclusion	• Ambient dehydration causing shrinkage, stiffening & drift • Loss of conductive contacts below percolation threshold
Molecular/hydration control handle	• Micro-phase segregation to optimize electronic/ionic pathways • Limiting hydration near fixed charges to enhance permselectivity	$•\;\mathrm{Lowering}\;\mathrm{hydration}\;\mathrm{number}\;(n_{h}$) to reduce the activity of bulk-like water • Regulating non-freezing bound water in nanopores	• Interpenetrating dual percolation networks • Organohydrogel hybridization (glycerol/ILs) to lock bound water
Main performance metric	$•\;\mathrm{Transconductance}\;(g_{m}$) • On/off switching speed • Faradaic efficiency	• Voltage window (>2–3 V) • Transference number ($t_{+}$) • Power density	• Sensitivity (S) • Gauge factor & hysteresis • Long-term signal stability
Sources	[1,32,42,79,91,93,94,97,98]	[4,9,10,31,32,37,38,53,54,56,76,99]	[1,19,34,43,48,49,50,51,52,100,101,102,103,104]

Abbreviations & Symbols: OECTs: Organic Electrochemical Transistors; OEIPs: Organic Electronic Ion Pumps; WiSE: Water-in-Salt Electrolyte; RED: Reverse Electrodialysis; ESW: Electrochemical Stability Window; ILs: Ionic Liquids; IPN: Interpenetrating Polymer Network; PEDOT:PSS: Poly(3,4-ethylenedioxythiophene):poly(styrenesulfonate); p(g2T-TT): Glycolated poly(thiophene-thienothiophene); qPVBC—Quaternized poly(vinylbenzyl chloride); PVBPPh3—Poly(vinylbenzyltriphenylphosphonium chloride); PSSS: Poly(sodium 4-styrenesulfonate); PVA: Poly(vinyl alcohol); PAAm: Polyacrylamide; PEO: Poly(ethylene oxide); SPEEK: Sulfonated poly(ether ether ketone); NaCMC: Sodium carboxymethyl cellulose; MXenes: Two-dimensional transition metal carbides/nitrides; $g_{m}$: Transconductance; $n_{h}$: Hydration number; $t_{+}$: Cation transference number; S: Sensitivity factor. Hydration must be optimised jointly with mechanical integrity. Additional water can facilitate ion motion but also change carrier concentration, network dimensions and resistance to deformation. Increasing crosslinking or introducing reinforcing phases can improve shape retention while modifying solvent uptake and the continuity of ion-conducting regions. The useful operating condition is therefore application-dependent and should be evaluated using conductivity and mechanical measurements on samples with the same conditioning history [1,5,48].

**Table 4:** Quantitative comparison of representative hydrated polymer electrolytes across transport archetypes, hydration regimes, and practical trade-offs.

System & Network	Hydration Regime/Water Content	Ionic Conductivity, σ (Measurement Temperature, *T*)	Dominant Mechanism; Activation Energy, $E_{a}$	Application & Practical Trade-Offs	Ref.
Charged Polyelectrolyte (AM-co-AMPS/APTAC)	Fully swollen equilibrium network; composition-dependent swelling	$2\cdot 10^{-4}$ –${8\cdot 10}^{-3}{\;S\;\mathrm{cm}}^{-1}$ (*T* n.r.)	Vehicular transport of Cl^−^ and H_3_O^+^; additional Grotthuss hopping of H^+^; $E_{a}$ n.r.	Biomimetic soft ionotronics; high mobility vs. osmotic swelling dilution of charges	[47]
WiPSE (PAAK/$K^{+}$)	Water-deficient polymer salt PAAK/H_2_O = 1:2 w/w $(H_{2}O/K^{+}=12$)	74 mS cm^−1^ at 25 °C	Hydrated K+ transport through aqueous domains, coupled to water diffusion; $E_{a}\approx 18.5{\;\mathrm{kJ}\;\mathrm{mol}}^{-1}$ (5–40 °C)	Large-scale supercapacitors; high conductivity and nonflammability, but water electrolysis limits practical operation to ≈2 V	[31]
Low-hydration SPE (PAGE/LiTFSI; 10–20 wt% LiTFSI)	0–80% RH; $w_{u}$ < 0.06 g H_2_O g^−1^ SPE (below water percolation)	${1\cdot 10}^{-5}$–${7\cdot 10}^{-5}{\;S\;\mathrm{cm}}^{-1}$ (${25\;}^{\circ }C$)	Preferential Li^+^ hydration weakens Li^+^–ether coordination; transition at $n_{H_{2}O}/n_{Li}\approx 4$; $E_{a}$ n.r.	Solid-state batteries and Li^+^ separations; hydration enhances Li^+^ mobility, but conductivity remains low and moisture-sensitive	[33]
PVA/PAM/LiCl double-network organohydrogel	Water/glycerol = 2:1 v/v; 2 M LiCl	Absolute σ n.r.; relative resistance measured over 20–80 °C	Li^+^ and Cl^−^ migration through the continuous water/glycerol phase; $E_{a}$ n.r.	Wearable strain, temperature, and respiratory sensors; stable from −20 to 60 °C, but with initial viscoelastic drift and temperature-cycle hysteresis	[51]
Boron-stabilized anisotropic PVA/LiTFSI WiPSE (1B-BaP)	0.5–4 m LiTFSI; internal water content n.r.	5.0–7.4 mS cm^−1^ at RT (0.5–2 m)	Li^+^ transport through aligned PVA channels; $E_{\alpha }(\sigma )\approx 12\;kJ{mol}^{-1}$ (4 m; 0–60 °C)	Aqueous LMO||LTO batteries; 3.09 V electrochemical window at 4 m, but ≥2 wt% borax causes brittleness	[56]
PVA–PSBMA (2:1) double-network hydrogel/PEDOT:PSS OECT	NaCl-swollen; EWC = 89.3%	6.0 ± 0.1 mS cm^−1^ at 1.0 M NaCl (*T* n.r.)	Na^+^ migration through the hydrated hydrogel, followed by interfacial desolvation and interchain hopping in PEDOT:PSS; *E*_*a*_ n.r.	Bioelectronic OECTs; τ = 2.0 ± 0.2 ms at 0.1 M NaCl; >96% current but 81.5% response speed retained after 1000 cycles	[105]

Abbreviations: EWC, equilibrium water content; OECT, organic electrochemical transistor; RH, relative humidity; RT, room temperature; WiPSE, water-in-polymer-salt electrolyte; m, molality (mol kg^−1^); n.r., not reported. Ea values refer to ionic conductivity. Reported conductivity values apply only to the stated composition, hydration state, and measurement temperature.

## Data Availability

No new data were created or analyzed in this study. Data sharing is not applicable to this article.
